# Behcet's Disease With Budd-Chiari Syndrome and Challenges in Its Management

**DOI:** 10.14309/crj.0000000000000352

**Published:** 2020-03-19

**Authors:** Sudheer K. Vuyyuru, Shivanand Gamanagatti, Shalimar

**Affiliations:** 1 Department of Gastroenterology, All India Institute of Medical Sciences, New Delhi, India; 2 Department of Radiodiagnosis, All India Institute of Medical Sciences, New Delhi, India

## Abstract

Budd-Chiari syndrome may rarely occur as a complication of Behcet's disease, and presentation with thrombosis of both inferior vena cava (IVC) and hepatic veins is rarer still. We present a young woman with Behcet's disease who presented with acute Budd-Chiari syndrome, with thrombosis of IVC and all 3 hepatic veins. An IVC stent was placed, followed by a transjugular intrahepatic portosystemic shunt through the IVC stent. On follow-up, despite oral anticoagulants and oral steroids, she developed recurrent thrombosis twice within a 1-year span. Her symptoms resolved with stent revision and increasing immunosuppression.

## INTRODUCTION

Behcet's disease (BD) is a multisystem vasculitis known to be complicated by vascular thrombosis.^1^ It is more common in Mediterranean countries and East Asia, along the silk route.^2^ Clinical presentation includes recurrent oral and genital ulcers, skin lesions, and arthritis. BD is rare in India; only 3 major case series and few case reports are available from India.^3,4^ BD is a rare cause of Budd-Chiari syndrome (BCS), responsible for less than 5% of cases but may be responsible for a higher proportion of cases in the endemic areas.^5,6^ The inferior vena cava (IVC) is most commonly involved. In one series of 1,200 patients with BD, isolated involvement of IVC and hepatic vein (HV) thrombosis was seen in 0.4% and 0.08% cases, respectively.^7^ In a review of 61 patients, 91% had IVC involvement.^8^ We present a case of BD with BCS and discuss management issues.

## CASE REPORT

A 19-year-old woman presented with abdominal distension due to ascites, followed by abdominal pain and pedal edema for 3 months duration. She was diagnosed with BD 2 years before the current presentation, based on the international criteria for BD and international society group criteria.^9,10^ Abdominal Doppler ultrasound showed IVC thrombus, along with thrombosis of all 3 HVs. She underwent IVC angioplasty twice within a span of 1 month and was started on oral anticoagulation in the form of warfarin (vitamin K antagonist). As no symptomatic improvement was achieved, IVC stenting was performed and she was referred to our center for further management.

The patient had tender hepatosplenomegaly and ascites. Investigations revealed elevated aspartate aminotransferase 407 IU/L, alanine aminotransferase 84 IU/L, and alkaline phosphatase 313 U/L. Ultrasound Doppler revealed thrombosis of all 3 HVs and patent IVC stent with adequate flow. Hypercoagulable workup revealed low protein C (49%). Antithrombin III, serum homocysteine, and protein S levels were within the normal range. Testing for antiphospholipid antibodies was negative. Other common causes of elevated transaminases, including viral hepatitis, autoimmune hepatitis, and Wilson disease, were ruled out.

**Figure 1.**
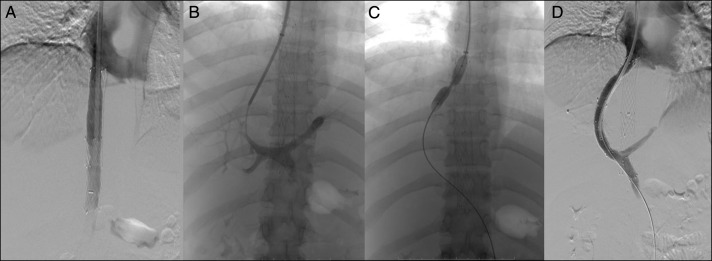
Placement of transjugular intrahepatic portosystemic shunt through the inferior vena cava stent by dilating struts with the balloon. (A) Inferior vena cavagram showing patent stent, (B) puncture of the portal vein with Ross Modified Colapinto Needle, (C) dilatation of struts of inferior vena cava stent with 6 mm × 4 cm balloon catheter, and (D) venogram showing complete diversion of portal blood to systemic circulation across the TIPS stent.

A transjugular intrahepatic portosystemic shunt (TIPS) was created to relieve the HV obstruction. The venogram of the IVC showed a patent stent. A combination of stents, including an uncovered self-expandable stent (10 mm ×10 cm) across the main portal vein and self-expandable covered stent (10 mm × 12 cm) across the hepatic parenchyma was placed. Post-TIPS venogram showed complete diversion of portal blood to the systemic circulation across the TIPS stent (Figure 1).

**Figure 2.**
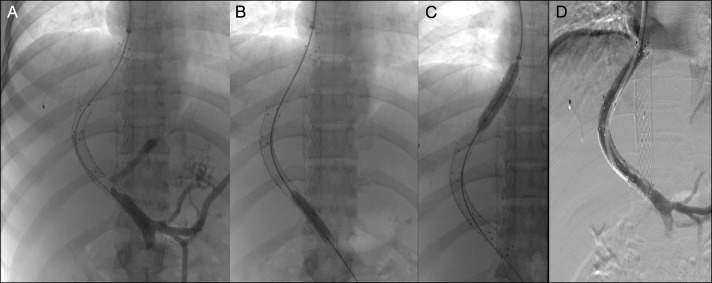
Technique showing revision of transjugular intrahepatic portosystemic shunt block by simple balloon dilatation. (A–C) Balloon angioplasty using 10 mm × 4 cm balloon catheter and (D) venogram showing the restoration of patency.

Postprocedure, the patient was stable and discharged on warfarin with a target international normalized ratio of 2–2.5. Two weeks postprocedure, there was complete resolution of ascites with complete normalization of transaminases. Three months later, she had a recurrence of ascites. A repeat Doppler showed a blocked TIPS stent. A balloon angioplasty (10 mm × 4 cm) was performed via a transjugular route. Postrevision venogram showed good decompression of portal blood flow across the TIPS stent (Figure 2). Although her international normalized ratio was in the therapeutic range, the occurrence of shunt obstruction prompted the initiation of oral steroids, which were tapered to the maintenance dose, to manage the underlying BD. She remained asymptomatic, and regular Doppler scans showed a patent stent. However, after 1 year of TIPS placement, she again developed a recurrence of stent thrombus. She also complained of arthralgias and had developed acne. Balloon angioplasty and a revision of the TIPS stent were performed. In view of disease activity, she was also given IV steroids, followed by an immunomodulator (azathioprine) in consultation with the rheumatologists.

## DISCUSSION

BD was first described by Hippocrates and later by Hulusi Behçet. It is a multisystemic disorder with dermatological, musculoskeletal, and vascular involvement. BCS in BD is a rare vascular complication with high mortality. Seyahi et al reported decompensated liver disease as the most common cause of death among patients with BCS-BD.^11^

Immunosuppression plays an important role in the management of BD with deep vein thrombosis.^12^ Glucocorticoids with either cyclophosphamide or azathioprine are recommended.^13^ In controlled trials, azathioprine has been shown to reduce the incidence of thrombosis.^14,15^ Anticoagulation may be beneficial when BD is associated with other prothrombotic states. Anticoagulants also play an important role postendovascular intervention in BCS to maintain patency of stents.^16^ Our patient had low protein C levels, which could be due to acute thrombotic events and warfarin, which our patient was already on. We could not confirm protein C deficiency because of the interference of oral anticoagulants with the measurements of protein C.

In the literature review of 61 cases of BD with BCS, only 3 patients were managed with endovascular treatment (one thrombolysis, one stenting, and one dilatation with stent). Most patients were managed with immunosuppressants and anticoagulants with a mortality of 34% (mean follow-up of 30 months).^8^ Radiological interventions are associated with better outcomes when compared with anticoagulation alone in patients with BCS.^17,18^ The placement of TIPS in a patient with already existing IVC stent is technically challenging. We broke the struts of the IVC stent to create an intrahepatic tract. We could find only one case report describing a similar intervention. Our patient had complete symptomatic resolution with normalization of transaminases post-TIPS, but stent thrombosis occurred twice within 1 year, despite adequate patient compliance with oral anticoagulants and steroids. The reason for frequent stent thrombosis could possibly be inadequate immunosuppression. After the last stent revision procedure, we increased her immunosuppression. She was symptom-free at the time of her last outpatient visit and remained on follow-up with us. The involvement of both HVs and IVC is rare in BD. Placement of TIPS through a pre-existing IVC stent is technically challenging. Immunosuppression is an essential component of the management of BD cases with BCS in addition to vascular interventions and anticoagulation.

## DISCLOSURES

Author contributions: SK Vuyyuru wrote the manuscript. S. Gamanagatti provided the radiological images. Shalimar revised the manuscript for intellectual content, approved the final manuscript and is the article guarantor.

Financial disclosure: None to report.

Informed consent was obtained for this case report.
